# Hyperpolarized ^13^C and ^31^P MRS detects differences in cardiac energetics, metabolism, and function in obesity, and responses following treatment

**DOI:** 10.1002/nbm.5206

**Published:** 2024-07-12

**Authors:** Andrew J. M. Lewis, Michael S. Dodd, Joevin Sourdon, Craig A. Lygate, Kieran Clarke, Stefan Neubauer, Damian J. Tyler, Oliver J. Rider

**Affiliations:** ^1^ Division of Cardiovascular Medicine, Radcliffe Department of Medicine University of Oxford Oxford UK; ^2^ Department of Physiology, Anatomy and Genetics University of Oxford Oxford UK; ^3^ Centre for Health and Life Sciences Coventry University Coventry UK

**Keywords:** diastolic function, hyperpolarized MRI, obesity

## Abstract

Obesity is associated with important changes in cardiac energetics and function, and an increased risk of adverse cardiovascular outcomes. Multi‐nuclear MRS and MRI techniques have the potential to provide a comprehensive non‐invasive assessment of cardiac metabolic perturbation in obesity. A rat model of obesity was created by high‐fat diet feeding. This model was characterized using in vivo hyperpolarized [1‐^13^C]pyruvate and [2‐^13^C]pyruvate MRS, echocardiography and perfused heart ^31^P MRS. Two groups of obese rats were subsequently treated with either caloric restriction or the glucagon‐like peptide‐1 analogue/agonist liraglutide, prior to reassessment. The model recapitulated cardiovascular consequences of human obesity, including mild left ventricular hypertrophy, and diastolic, but not systolic, dysfunction. Hyperpolarized ^13^C and ^31^P MRS demonstrated that obesity was associated with reduced myocardial pyruvate dehydrogenase flux, altered cardiac tricarboxylic acid (TCA) cycle metabolism, and impaired myocardial energetic status (lower phosphocreatine to adenosine triphosphate ratio and impaired cardiac Δ*G*_~ATP_). Both caloric restriction and liraglutide treatment were associated with normalization of metabolic changes, alongside improvement in cardiac diastolic function. In this model of obesity, hyperpolarized ^13^C and ^31^P MRS demonstrated abnormalities in cardiac metabolism at multiple levels, including myocardial substrate selection, TCA cycle, and high‐energy phosphorus metabolism. Metabolic changes were linked with impairment of diastolic function and were reversed in concert following either caloric restriction or liraglutide treatment. With hyperpolarized ^13^C and ^31^P techniques now available for human use, the findings support a role for multi‐nuclear MRS in the development of new therapies for obesity.

## Graphical abstract

Integrated complementary multi‐nuclear MRS techniques were employed to detect cardiac energy metabolism changes at various levels in an obese rat model. Utilizing hyperpolarized [1‐^13^C]pyruvate, hyperpolarized [2‐^13^C]pyruvate, and ^31^P cardiac MRS, the study demonstrated the potential for assessing and detecting treatment responses, highlighting clinical translational possibilities.

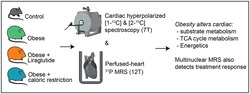

**Abbreviations**
*E*/*e*′: ratio between early mitral inflow velocity and mitral annular early diastolic velocity
GLP‐1: glucagon‐like peptide‐1
HFD: high‐fat diet
kcal: kilocalorie
LV: left ventricle
PCr/ATP: ratio of phosphocreatine to adenosine triphosphate;
PDH: pyruvate dehydrogenase
SD: standard diet
TCA: tricarboxylic acid
*T*_R_
: repetition time (in the context of magnetic resonance)

## 1 INTRODUCTION

Obesity is associated with significant changes in the structure and function of the heart, and an increased risk of heart failure^1^ and cardiovascular morbidity/mortality.^2^, ^3^ Mechanisms underlying cardiac dysfunction and increased cardiovascular risk in obesity are not well understood but are likely to include altered hemodynamic conditions, low‐grade inflammation, and in particular abnormal myocardial energy metabolism.^4^, ^5^, ^6^ As a result, there is currently significant interest in developing new treatments that could modulate cardiac metabolism, function, and risk in obesity.^7^, ^8^, ^9^ Non‐invasive imaging tools that can probe disease mechanisms and assess treatment responses have an important role in the clinical development of such emerging therapies.^8^, ^10^

Proton‐based cardiovascular magnetic resonance (CMR) techniques are well optimized and provide a gold‐standard assessment of the structure and function of the heart.^11^ Hyperpolarized MR is a newer technology, which generates molecular contrast agents with an improvement in MR signal of up to five orders of magnitude.^12^, ^13^ By administering hyperpolarized [1‐^13^C]pyruvate and [2‐^13^C]pyruvate, it is possible to probe myocardial metabolic substrate selection (via pyruvate dehydrogenase (PDH) flux) and metabolism through the first span of the tricarboxylic acid (TCA) cycle^14^, ^15^ (Figure 4 later). ^31^P spectroscopy is a more established MR tool, which can probe cardiac high‐energy phosphorus metabolism for assessment of energetic deficits that may link altered cardiac metabolism to impaired cardiac function.^16^

We hypothesized that obesity would be associated with abnormal cardiac energy metabolism and function, and that cardiac metabolic perturbations could be assessed at multiple levels by combining hyperpolarized ^13^C and ^31^P spectroscopy. We also hypothesized that multi‐nuclear MR approaches might have utility for the detection of responses to treatment. In this study, we created a rat model of obesity, characterized cardiac energy metabolism using hyperpolarized ^13^C and ^31^P spectroscopy, and assessed for changes in cardiac function and metabolism following treatment with either the glucagon‐like peptide (GLP)‐1 analogue/agonist liraglutide (for 7 d) or caloric restriction (for 28 d).

## 2 METHODS

### 2.1 Animal model

**FIGURE 1:**
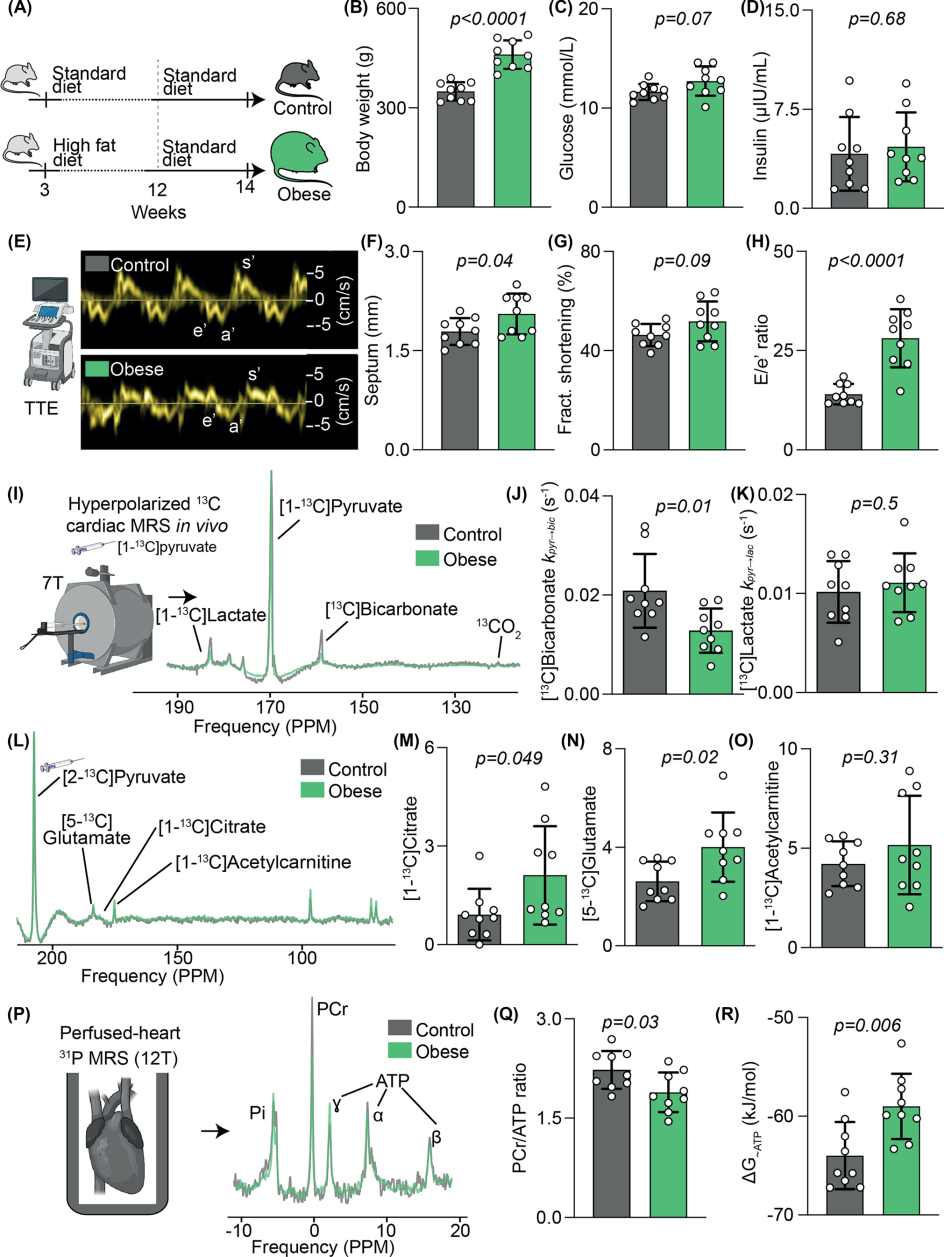
Characterization of a model of diet induced obesity. A, Experimental model; B, body weight; C, plasma glucose; D, plasma insulin; E, echocardiography with representative tissue Doppler traces; F, LV wall thickness measured at the septum; G, LV systolic function assessed using fractional shortening; H, diastolic function assessed using *E*/*e*′; I, hyperpolarized [1‐^13^C]pyruvate spectroscopy; J, ^13^C label incorporation into [1‐^13^C]bicarbonate as *K*_pyr‐bic_; K, ^13^C label incorporation into [1‐^13^C]lactate as *K*_pyr‐lac_; L, hyperpolarized [2‐^13^C]pyruvate spectroscopy; M, normalized [1‐^13^C]citrate signal; N, normalized [5‐^13^C]glutamate signal; O, normalized [1‐^13^C]acetylcarnitine signal; P, perfused heart ^31^P spectroscopy with representative spectra; Q, PCr/ATP; R, free energy change of ATP hydrolysis.

All procedures were carried out in accordance with the UK Animals (Scientific Procedures) Act 1986. Outbred male Long Evans (LE) rats with diet induced obesity and lean controls were purchased from Harlan Laboratories, Indianapolis, IN, USA. In this model, obesity was induced by high‐fat diet (HFD) from weaning at the age of 3 weeks, until at least 12 weeks (Figure 1, Teklad Custom Research Diet TD.06414, 60% kilocalories (kcal) from fat, 18% kcal from protein, 21% kcal from carbohydrate ad libitum). Control animals (male outbred LE) were maintained on a standard diet (SD, Teklad Global Diet 2014S, 13% kcal from fat, 20% kcal from protein, 67% kcal from carbohydrate ad libitum). All animals were standardized to normal diet (2014S) for at least 5 d prior to baseline assessment to reduce the potentially confounding effect of HFD upon metabolism.^17^

One group of rats (*n* = 9) underwent study assessments at 14 weeks after completion of the HFD/diet standardization protocol to characterize the obesity model. Another group of obese rats underwent the same protocol (*n* = 9) followed by treatment with liraglutide 0.2 mg/kg by subcutaneous injection twice daily for 7 d. A further group of obese rats (*n* = 9) underwent caloric restriction to 70% of the usual intake of SD for 28 days. Comparisons from both treatment groups were made with the pre‐treatment obese group.

Both of these groups were then reassessed following treatment completion.

### 2.2 Echocardiography

Echocardiography was performed under light isoflurane anaesthesia with oxygen as a carrier gas using a Vivid I ultrasound scanner with 11.5 MHz 10S‐RS probe (both from GE Healthcare, Chicago, USA). Systolic function was assessed from parasternal M‐mode views. Diastolic function was assessed using pulsed wave Doppler examination directed to the mitral valve inflow (E wave) and tissue Doppler imaging of the medial mitral annulus (to derive *e*′). Analysis was performed using a commercially available software package (Xcelera, Philips Healthcare, Andover, MA, USA) by an expert operator blinded to the experimental group.

### 2.3 Hyperpolarized [1‐^13^C] and [2‐^13^C]pyruvate MRS

Hyperpolarized [1‐^13^C] and [2‐^13^C]pyruvate MRS experiments were performed as previously described.^18^ Rats in the fed state (between 7 AM and 1 PM) were anaesthetized with 2.5% isoflurane in oxygen and positioned in a 7.0 T horizontal bore MR system (Varian, Yarnton, UK) scanner interfaced to a Direct Drive console (Varian), and a home‐built 1H/13C butterfly coil (loop diameter 2 cm) was placed over the chest, localizing the signal from the heart. Correct positioning was confirmed by the acquisition of an axial proton FLASH image (echo time 1.17 ms; repetition time 2.33 ms; matrix size 64 × 64; field of view 60 mm × 60 mm; slice thickness 2.5 mm; excitation flip angle 15°). An electrocardiogram‐gated shim was used to reduce the proton linewidth to about 120 Hz. Hyperpolarized pyruvate was generated using about 40 mg of either [1‐^13^C]pyruvic acid or [2‐^13^C]pyruvic acid doped with 15 mM trityl radical (OXO63, Oxford Instruments, Abingdon, UK) and 3 μL Dotarem (1:50 dilution, Guerbet, Birmingham, UK) in a prototype polarizer system, with 45 min of microwave irradiation. The sample was subsequently dissolved in a pressurised and heated alkaline solution, containing 2.4 g/L sodium hydroxide and 100 mg/L EDTA dipotassium salt (Sigma‐Aldrich), to yield a solution of 80 mM hyperpolarized sodium [1‐^13^C]pyruvate or [2‐^13^C]pyruvate with a polarization of about 30 or about 20%, respectively, at physiological temperature and pH.

Immediately following dissolution, 1 mL of the ^13^C hyperpolarized substrate was injected intravenously into the anaesthetized animal over 10 s. Sixty individual electrocardiogram‐gated ^13^C MR slice‐selective, pulse‐acquired cardiac spectra were acquired over 60 s after injection (repetition time 1 s; excitation flip angle 5°; slice thickness 10 mm; sweep width 13 593 Hz; acquired points 2048; frequency centred on the C1 pyruvate resonance). The combination of surface coil localization with slice selection ensured that the signals measured were accurately localized to the cardiac lumen (pyruvate) and the front wall of the myocardium (downstream metabolites). Minimal contribution was provided from the skeletal muscle because of the large differential in metabolic rate between the contracting myocardium and the resting skeletal muscle.

All cardiac ^13^C spectra were analysed using the algorithm advanced method for accurate, robust, and efficient spectral fitting (AMARES) in the jMRUI software package. Spectra were direct current offset corrected based on the last half of acquired points. For hyperpolarized [1‐^13^C]pyruvate, spectra were fitted to a kinetic model specifically designed to assess rates of label incorporation from hyperpolarized pyruvate metabolism.^19^, ^20^ For hyperpolarized [2‐^13^C]pyruvate, spectra were summed for the first 60 s following appearance of hyperpolarized pyruvate, with the peak areas of [1‐^13^C]acetylcarnitine, [1‐^13^C]citrate, and [5‐^13^C]glutamate quantified following the injection of [2‐^13^C]pyruvate. Metabolite peak areas were normalized to the peak area of hyperpolarized [2‐^13^C]pyruvate to account for any differences in absolute polarization level. These ratios were then also normalized to the rate of [1‐^13^C]pyruvate label in [^13^C]bicarbonate (which reflects PDH flux), as PDH flux controls the rate of entry of [2‐^13^C]pyruvate label propagation into downstream metabolites.

### 2.4 ^31^P spectroscopy

The ^31^P experiments were conducted on an 11.7 T (500 MHz) MR system comprising a vertical bore magnet (Magnex Scientific, Yarnton, Oxford, UK) and a Bruker Avance console (Bruker Medical, Ettlingen, Germany). A dual‐tuned birdcage coil with an inner diameter of 20 mm (Rapid Biomedical, Wurzburg, Germany) was used as a transmit/receive coil for both phosphorus and protons. The hearts were cannulated via the aorta and perfused in Langendorff mode with modified Krebs–Henseleit buffer at 37  C under constant perfusion pressure conditions. End diastolic pressure was set to 5–10 mmHg. Left ventricular (LV) pressure was monitored throughout the experiment with a balloon inserted into the left ventricle and connected via a polyethylene tube to a bridge amplifier and PowerLab data acquisition system (ADInstruments, Oxfordshire, UK). The balloon volume was adjusted to achieve an initial end diastolic pressure of 5–10 mmHg. The heart was lowered into the centre of the magnet and the position verified with proton scout images. After shimming, the water resonance linewidth was less than 50 Hz. ^31^P MR spectra were acquired at 202.5 MHz using a 90° RF pulse and a repetition delay of 15 s. The phosphocreatine (PCr) resonance was set at 0 ppm and the chemical shifts of all peaks were referenced to that of PCr. Each spectrum consisted of 40 transients, giving a total acquisition time of 10 min. ^31^P MR spectra were analysed using the AMARES algorithm in the jMRUI software package. Spectra were corrected for any DC offset using the last half of acquired points. The PCr, P_i_, and α‐, β‐, and γ‐ATP resonances were fitted assuming Lorentzian line shapes, peak frequencies, relative phases, linewidths, and *J*‐coupling parameters. The free energy change of ATP hydrolysis, Δ*G*_~ATP_, was derived according to the expression Δ*G*_~ATP_ = Δ*G* ^0^_~ATP_ − *RT* ln [ATP]/[ADP][P_i_]. [ADP] was derived from the creatine kinase equilibrium equation [ADP] = [ATP][Creatine]/*K*_eq_[PCr][H^+^], whilst [H^+^] was determined from the chemical shift of inorganic phosphate. As myocardial ATP levels are tightly regulated and begin to fall only after the development of advanced heart failure, ATP levels were assumed to be constant (11 mM) between groups.^16^ Following ^31^P spectroscopy, hearts were snap frozen on the perfusion cannula using aluminium tongs cooled to the temperature of liquid nitrogen for subsequent analysis. The myocardial total creatine pool size was assessed using high‐performance liquid chromatography (HPLC) as previously described,^21^ normalized to non‐collagenous protein content and expressed as mmol/L intracellular water.

### 2.5 Plasma metabolite measurement

Blood samples were collected from the thoracic cavity following isolation of the heart and a 50 μL aliquot of plasma separated to measure plasma glucose in an automated assay system (Pentra 400, Horiba ABX Diagnostics, Montpellier, France). A separate aliquot of 10 μL was used in a commercially available enzyme linked immunosorbent assay for quantitative determination of insulin (Mercodia, Uppsala, Sweden).

### 2.6 Statistical analysis

The data are reported as mean ± standard deviation. After evaluating the normality of the distribution, statistical comparisons were conducted using two‐tailed, unpaired *t*‐tests or their non‐parametric counterparts. A significance threshold of *p* ≤ 0.05 was applied to determine statistical significance. The data that support the findings of this study will be made available from the corresponding author upon reasonable request; study data are also presented in a tabular format in Table S1.

## 3 RESULTS

### 3.1 Characterization of the cardiac phenotype in a rat model of obesity using MRS

HFD for 12 weeks was associated with a significant 23% increase in body weight (Figure 1A,B) but no difference in plasma glucose (Figure 1C) or insulin (Figure 1D). Transthoracic echocardiography revealed that obese rats had a slight though significant increase in LV wall thickness (Figure 1E,F), mildly hyperdynamic systolic function (Figure 1G), and evidence of significant LV diastolic dysfunction, with a near doubling of the ratio between early mitral inflow velocity and mitral annular early diastolic velocity (*E*/*e*′, Figure 1H).

**FIGURE 2:**
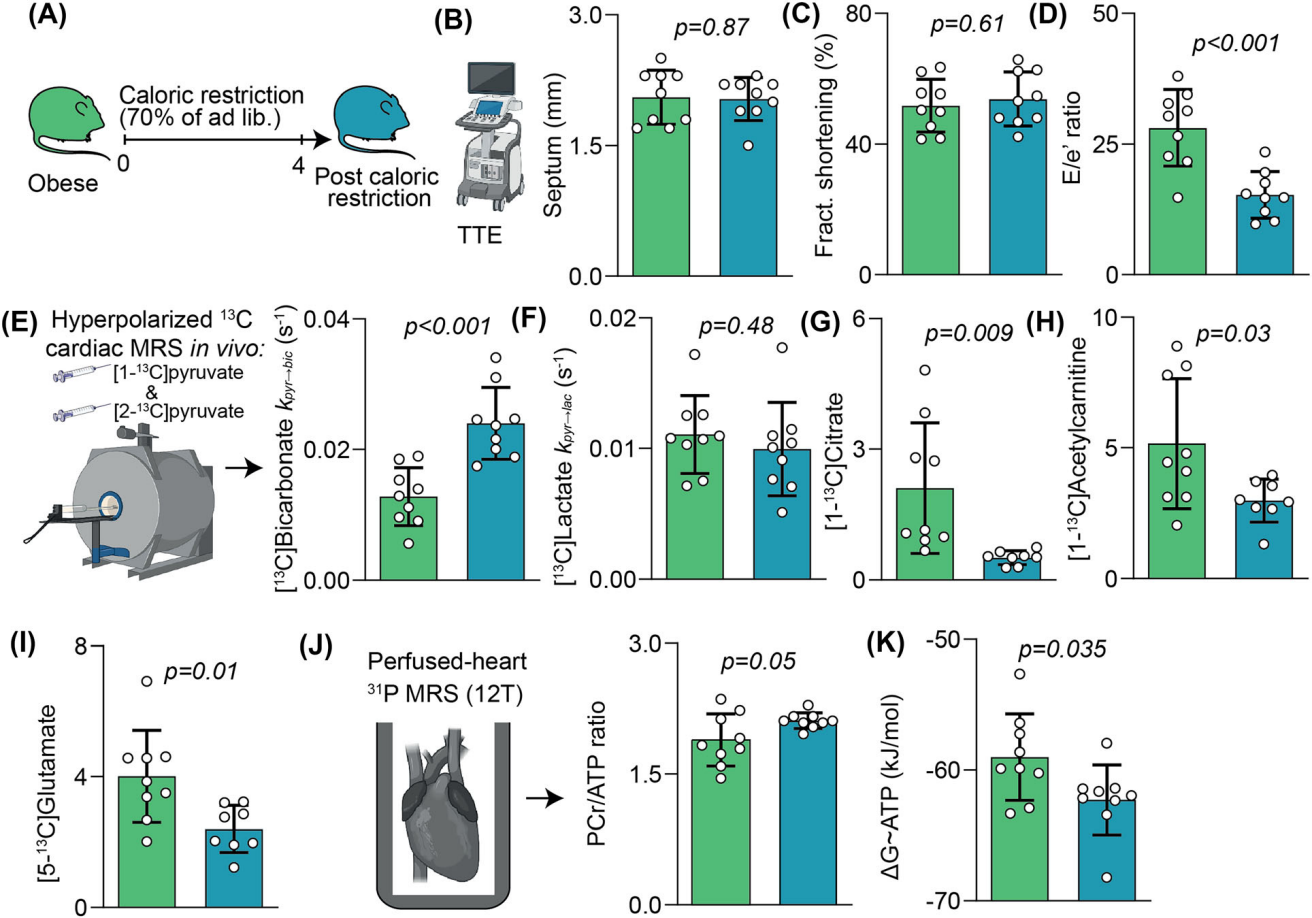
Assessment of treatment response following caloric restriction. A, Caloric restriction protocol; B, septal wall thickness; C, LV fractional shortening; D, diastolic function assessed from *E*/*e*′; E, hyperpolarized [1‐^13^C]pyruvate and [2‐^13^C]pyruvate spectroscopy with ^13^C label incorporation into [1‐^13^C]bicarbonate as *K*_pyr‐bic_; F, ^13^C label incorporation into [1‐^13^C]lactate as *K*_pyr‐lac_; G, normalized [1‐^13^C]citrate signal; H, normalized [1‐^13^C]acetylcarnitine signal; I, normalized [5‐^13^C]glutamate signal; J, perfused heart ^31^P spectroscopy with PCr/ATP; K, free energy change of ATP hydrolysis.

Hyperpolarized ^13^C MRS following an intravenous bolus injection of [1‐^13^C]pyruvate (Figure 1I) revealed that obesity was associated with a 40% reduction in the rate of ^13^C label incorporation into [^13^C]bicarbonate (i.e., PDH flux) compared with controls (Figure 1J). There were no significant differences in the rates of label incorporation from [1‐^13^C]pyruvate into either [1‐^13^C]lactate (Figure 1K) or [1‐^13^C]alanine (not shown). Following intravenous injection of hyperpolarized [2‐^13^C]pyruvate, quantifiable ^13^C label incorporation into [1‐^13^C]citrate, [5‐^15^C]glutamate, and [1‐^13^C]acetylcarnitine was detected (Figure 2L). Since [2‐^13^C]pyruvate must pass through the PDH complex to enter the TCA cycle (illustrated in Figure 4 later), and given that PDH flux was reduced in this model, these [2‐^13^C]pyruvate label incorporation rates were normalized for PDH flux in each rat. Compared with controls, obesity almost doubled the normalized rate of ^13^C label incorporation into [1‐^13^C]citrate (Figure 1M) and increased label incorporation into [5‐^15^C]glutamate by over 50% (Figure 1N). In contrast, there was no significant change in the normalized rate of label incorporation into [1‐^13^C]acetylcarnitine (Figure 1O).

^31^P spectroscopy was next used to assess the energetics of isolated perfused hearts (Figure 1P). Although there was no statistically significant difference in cardiac work (rate–pressure product 34 000 ± 5000 in obesity versus 30 000 ± 5000 mmHg·min^−1^ in control, *p* = 0.11), hearts from obese rats demonstrated lower energetic reserve, with a significant approximately 10% reduction in ratio of phosphocreatine to adenosine triphosphate (PCr/ATP; Figure 1Q), and significant approximately 8% change in cardiac Δ*G*_~ATP_ (Figure 1R, with Δ*G*_~ATP_ representing the chemical driving force available from ATP hydrolysis).

### 3.2 Assessment of myocardial metabolic and functional changes following caloric restriction

Caloric restriction of obese rats for 4 weeks (Figure 2A) was not associated with any significant change in body weight when compared with rats at the pre‐caloric restriction timepoint, due to normal body growth with ageing (505 ± 40 versus 505 ± 32 g, *p* = 0.16 by paired *t*‐test). However, ad libitum fed LE rats gain approximately 15%–20% body weight between weeks 14 and 18 of life,^22^ and so the failure to gain body weight during the CR period in our study probably indicates meaningful caloric restriction. Furthermore, the mean body weight of obese rats after caloric restriction was significantly lower than for a separate cohort of age‐matched obese rats (505 ± 9 versus 600 ± 10 g, *p* < 0.001), consistent with meaningful fat loss.

Caloric restriction was associated with no significant change in LV wall thickness (Figure 2B) or systolic function (Figure 2C), though LV diastolic function was significantly improved compared with pre‐treatment (46% reduction in LV *E*/*e*′, Figure 2D). Hyperpolarized MRS revealed that caloric restriction was also associated with near‐normalization of myocardial PDH flux (Figure 2E), with no change in label incorporation into [1‐^13^C]lactate (Figure 2F) or [1‐^13^C]alanine (not shown). There was a reduction in normalized label flux rates into [1‐^13^C]citrate (Figure 2G), [1‐^13^C]acetylcarnitine (Figure 2H), and [5‐^15^C]glutamate (Figure 2I). These metabolic and functional changes were associated with improvements in cardiac energetic reserve, assessed using both the PCr/ATP ratio (Figure 2J) and ΔG_~ATP_ (Figure 2K).

### 3.3 Assessment of myocardial metabolic changes following treatment with the GLP‐1 analogue liraglutide

Treatment with the GLP‐1 analogue liraglutide was not associated with significant weight loss over the one‐week treatment period (453 ± 36 versus 467 ± 37 g pre‐treatment, *p* = 0.43).

**FIGURE 3:**
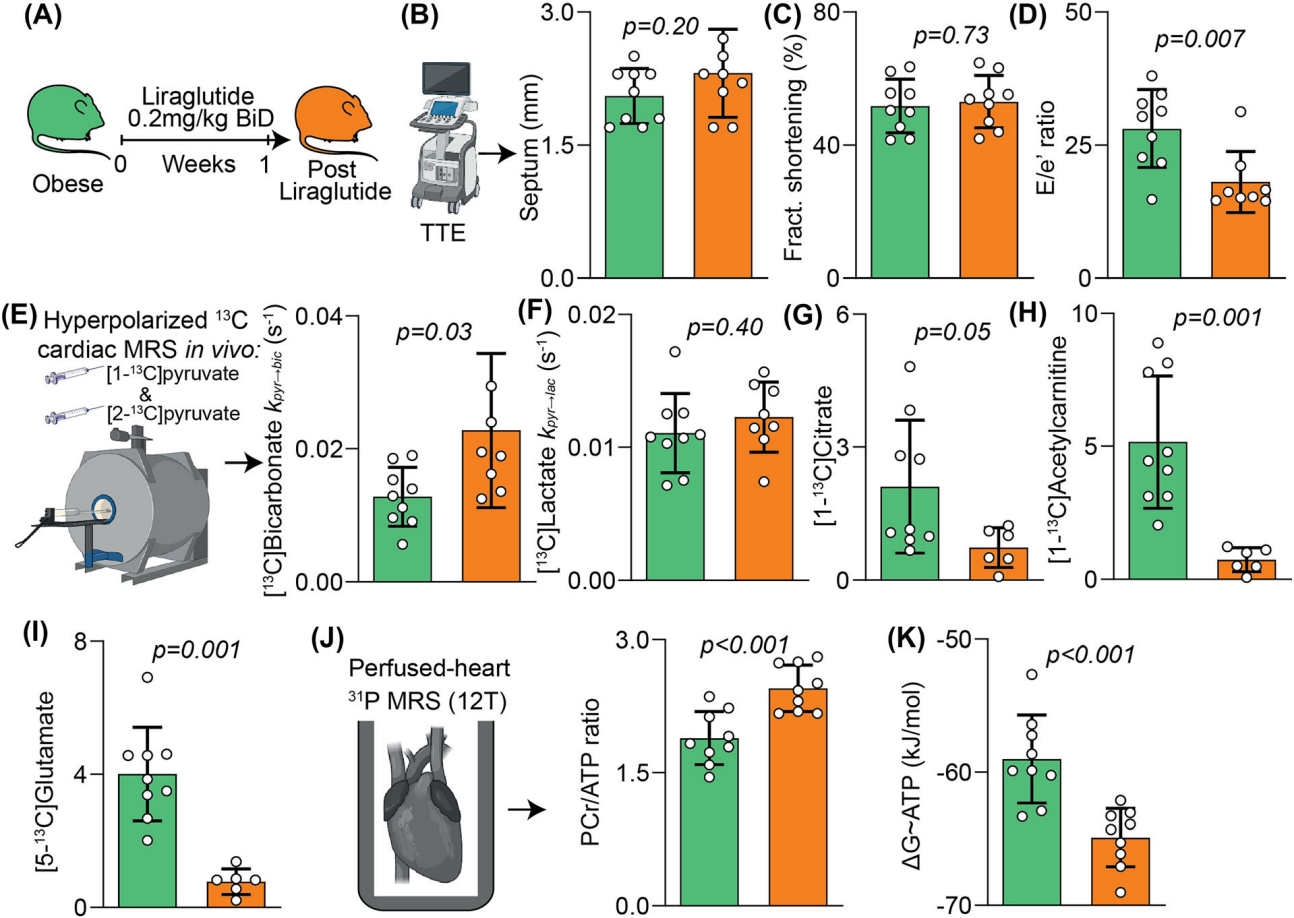
Assessment of treatment response following administration of the GLP‐1 analogue liraglutide. A, Caloric restriction protocol; B, septal wall thickness; C, LV fractional shortening; D, diastolic function assessed from *E*/*e*′; E, hyperpolarized [1‐^13^C]pyruvate and [2‐^13^C]pyruvate spectroscopy with ^13^C label incorporation into [1‐^13^C]bicarbonate as *K*_pyr‐bic_; F, ^13^C label incorporation into [1‐^13^C]lactate as *K*_pyr‐lac_; G, normalized [1‐^13^C]citrate signal; H, normalized [1‐^13^C]acetylcarnitine signal; I, normalized [5‐^13^C]glutamate signal; J, perfused heart ^31^P spectroscopy with PCr/ATP; K, free energy change of ATP hydrolysis.

Liraglutide treatment was associated with no change in LV wall thickness (Figure 3B) or systolic function (Figure 3C), though LV diastolic function was significantly improved (35% reduction in LV *E*/*e*′, Figure 3D).

When compared with the pre‐treatment obese group, ^13^C label incorporation into [^13^C]bicarbonate (i.e., PDH flux) was significantly increased after liraglutide treatment (Figure 3E) with no change in label incorporation into [1‐^13^C]lactate (Figure 3F) or [1‐^13^C]alanine (not shown). Liraglutide treatment was also associated with a reduction in normalized label flux rates into [1‐^13^C]citrate, [1‐^13^C]acetylcarnitine, and [5‐^15^C]glutamate (Figure 3G–I). This was paralleled by a significant improvement in cardiac energetic reserve (29% increase in PCr/ATP ratio, Figure 3J).

## 4 DISCUSSION

**FIGURE 4:**
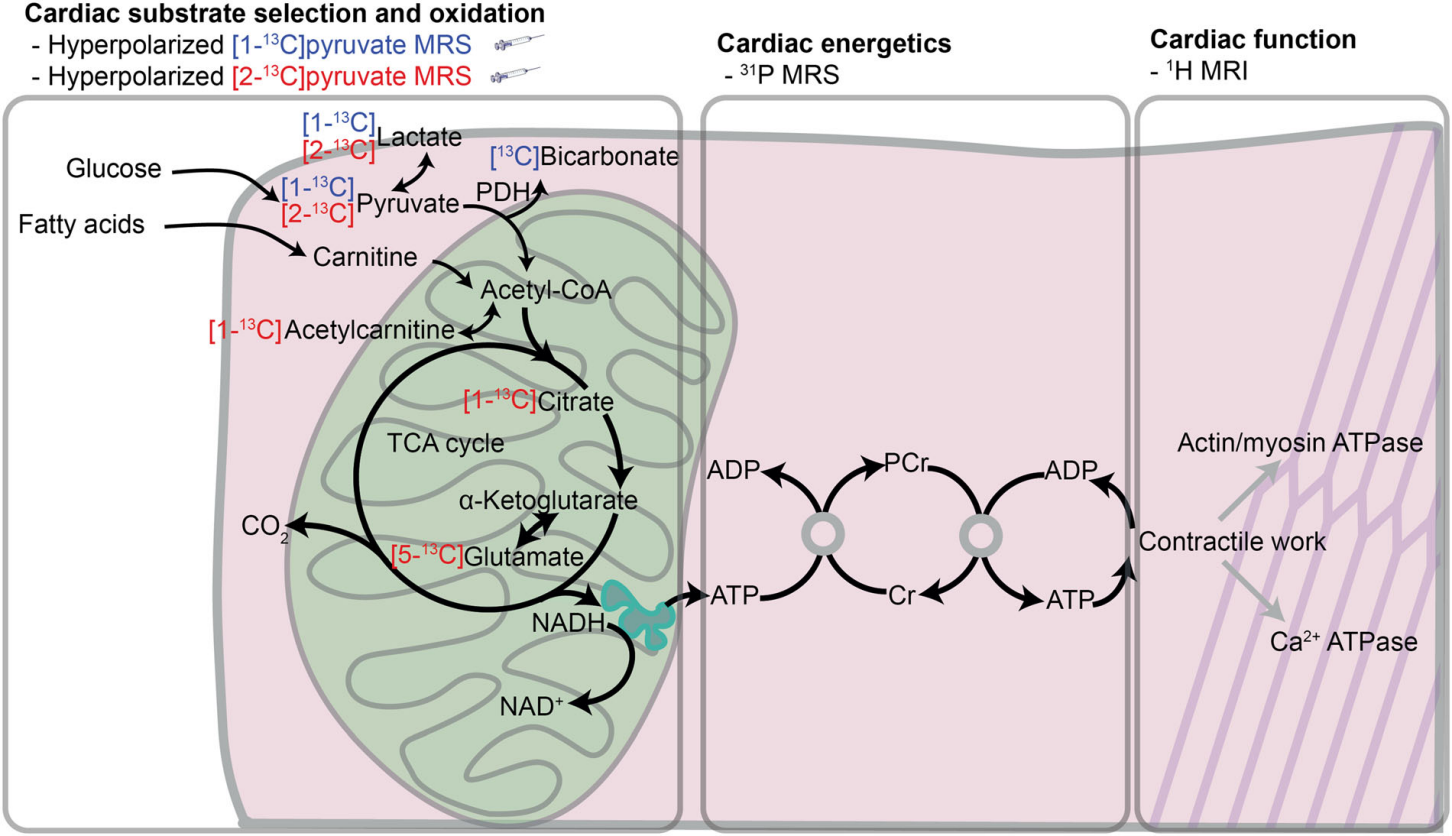
Overview of proposed assessment of myocardial metabolism, energetics, and function at multiple levels using hyperpolarized ^13^C MR including label propagations illustrating complementary roles of [1‐^13^C]pyruvate and [2‐^13^C]pyruvate, ^31^P spectroscopy, and ^1^H MRI. Acetyl‐CoA, acetyl‐coenzyme A; ADP, adenosine diphosphate; Cr, creatine; NADH, nicotinamide adenine dinucleotide (reduced form); NAD^+^ nicotinamide adenine dinucleotide (oxidized form); PDH, pyruvate dehydrogenase.

In this rat model of obesity, we used a combination of MR techniques including hyperpolarized [1‐^13^C]pyruvate and [2‐^13^C]pyruvate, and ^31^P spectroscopy to document changes in cardiac energy metabolism in obesity at multiple stages of energy generation (Figure 4). The same MR techniques detected metabolic responses following either treatment with caloric restriction or treatment with the GLP‐1 analogue liraglutide, and these metabolic changes were associated with cardiac functional improvement, suggesting physiological relevance.

Abnormal energy metabolism has long been considered a potential pathophysiological mechanism in obesity related heart disease, though assessment of cardiac metabolism non‐invasively is challenging. One advantage of hyperpolarized MRS is the ability to measure cardiac carbohydrate metabolism in vivo in conditions of physiological substrate availability. The reduction in PDH flux seen here is consistent with a reduction in cardiac glucose oxidation in obesity, which has also been documented following high‐fat feeding,^23^ and in diabetes.^14^, ^24^ Hyperpolarized [2‐^13^C]pyruvate MRS experiments probed TCA cycle metabolism in vivo, and obesity was associated with increased normalized rates of label incorporation into citrate and glutamate. There are several possible mechanisms that could explain these changes, including increased metabolite pool sizes, increased TCA cycle flux, or increased flux through enzymes including citrate synthase and others.^14^, ^25^ Additional research using complementary techniques is needed to better understand the mechanistic basis for signal changes in cardiac hyperpolarized [2‐^13^C]pyruvate MRS experiments.

Dietary caloric restriction was associated with favourable changes in cardiac energy metabolism and diastolic function in this rat model, and ongoing randomized clinical trials will assess whether intentional weight loss is beneficial in humans with obesity and heart failure. Several GLP‐1 analogues/agonists are also now available for the pharmacological treatment of obesity,^26^ and can improve symptoms in patients with heart failure with preserved ejection fraction.^27^ Newer GLP‐1 analogues/agonists cause a greater degree of weight loss than does liraglutide.^26^, ^28^ In our study, significant changes in cardiac metabolism were observed over a one‐week treatment period in which a significant reduction in body weight did not occur (although the failure to gain weight as would be expected at this age should also be considered). Future studies should therefore consider assessment at serial timepoints to separate weight‐neutral and weight‐loss related effects of GLP‐1 treatment. Previous studies have documented multiple pathways that might mediate beneficial cardiovascular effects of this class of medicine.^29^

The findings of this study may have translational potential, as both calorie restriction and GLP‐1 analogues/agonists are widely available. However, the optimal treatment strategies to modulate cardiac metabolism and function in obesity related heart disease are unknown. The clinical development of hyperpolarized ^13^C MRI and MRS^24^, ^30^ and ultra‐high‐field ^31^P spectroscopy^31^ could provide biomarkers for mechanistic and dose‐ranging studies of pharmacologic and non‐pharmacologic interventions in obesity and other forms of metabolic heart disease. Impaired cardiac energetic reserve may contribute to the depletion in PCr and further impairment of diastolic function observed during pharmacological stress in humans with obesity.^32^ Whether strategies to improve cardiac energetics and diastolic function would improve clinical outcomes in obesity remains unknown; however, diastolic dysfunction has been independently linked with adverse prognosis in other disease states.^33^

Our work had some limitations. We used echocardiography rather than ^1^H MRI to assess cardiac systolic function as we anticipated from previous data that major changes in systolic function were unlikely, and because echocardiography is preferable for assessing diastolic function. Since cardiac tissue underwent a Langendorff perfusion protocol for ^31^P spectroscopy, it could not be used for additional laboratory analyses with complementary techniques (such as metabolomics) in order to gain a more granular understanding of metabolic perturbations, though this could be tested in future studies. Liraglutide is now used less frequently, as newer GLP‐1 analogues/agonists, which cause a greater degree of weight loss (e.g., semaglutide, tirzepatide, and others), are now available. A longer duration of treatment with liraglutide would probably have led to weight loss and future studies should investigate the effect of duration of treatment to isolate weight‐loss‐dependent and weight‐loss‐independent effects of this drug. We studied only male rats in this work, which limits the generalizability of the study, due to important sex differences in the rate of weight gain in this specific strain of rat, which could have created an experimental confound. Future work should investigate whether there are sex differences in cardiac energy metabolism in animal models, and these imaging tools will probably be useful in future clinical studies, which should be designed to be representative of age, sex, ethnicity, and other factors. Since experimental groups were not age matched at the time of post‐treatment assessment, we cannot directly statistically compare the CR versus liraglutide groups, but this should be investigated in future studies. Given the need to normalize [2‐^13^C]pyruvate metabolite signals to PDH flux to control for the rate of label entry, we acknowledge that changes in both TCA cycle metabolism and PDH flux will probably affect these point estimates. Orthogonal techniques to isolate TCA cycle kinetics would be valuable in future studies.

## 5 CONCLUSIONS

Multi‐nuclear MR using hyperpolarized ^13^C MR and ^31^P spectroscopy detected changes in myocardial metabolism and energetics at multiple levels in this rat model of obesity related heart disease. These metabolic changes were linked to a reduction in cardiac diastolic function and were ameliorated in concert following treatment with either the GLP‐1 analogue/agonist liraglutide or caloric restriction. Our findings therefore suggest that non‐invasive multi‐nuclear MRS techniques may be valuable to understand the mechanisms of cardiac impairment in obesity, and for the clinical development of new therapies to treat cardiovascular consequences arising from obesity.

## CONFLICT OF INTEREST STATEMENT

Our laboratory has received equipment support from GE Healthcare. Otherwise, the authors have no relevant conflicts of interest to disclose.

## Supporting information


**Table S1.** Experimental parameters presented as mean ± SD.
