# Supplementary material for: Hyperpolarized 13C and 31P MRS detects differences in cardiac energetics, metabolism, and function in obesity, and responses following treatment
Source: NMR Biomed. 2024 Jul 12;37(11):e5206. doi: 10.1002/nbm.5206 (PMC11571269; doi:10.1002/nbm.5206)
Supplement: Supplementary file 1 — Table S1. Experimental parameters presented as mean ± SD. [file NBM-37-e5206-s001.docx]

**Supplemental table 1**

|  | **Control** | **Obese** | **Post liraglutide** | **Post CR** |
| --- | --- | --- | --- | --- |
| **Age at time of MRI assessment (weeks)** | 14 | 14 | 15 | 18 |
| **Body weight at time of MRI assessment (g)** | 350 ± 28 | 460 ± 43 | 453 ± 36 | 503 ± 32 |
| **Glucose (mmol/L)** | 11.6 ± 1 | 12.7 ± 2 | 12.8 ± 3 | 12.5 ± 1 |
| **Insulin (µIU/mL)** | 4.1 ± 3 | 4.7 ± 3 | 4.4 ± 2 | 3.1 ± 1 |
| **Septum (mm)** | 1.8 ± 0.2 | 2.1 ± 0.3 | 2.3 ± 0.5 | 2.0 ± 0.2 |
| **Fract shortening (%)** | 46 ± 4 | 52 ± 8 | 53 ± 8 | 54 ± 8 |
| **E/e' ratio** | 14 ± 3 | 28 ± 7 | 15 ± 41 | 15 ± 4 |
| **Left ventricular creatine (total creatine, ng/mg protein)** | 103 ± 7 | 125 ± 11 | 128 ± 28 | 109 ± 41 |
| **[1-^13^C]bicarbonate (K_pyr-bic_, s^-1^)** | 0.021 ± 0.01 | 0.013 ± 0.004 | 0.023 ± 0.01 | 0.024 ± 0.01 |
| **[1-^13^C]lactate (K_pyr-lac_, s^-1^)** | 0.010 ± 0.003 | 0.011 ± 0.003 | 0.012 ± 0.003 | 0.010 ± 0.004 |
| **Norm. [1-^13^C]citrate** | 0.91 ± 0.8 | 2.11 ± 1.5 | 0.73 ± 0.4 | 0.51 ± 0.2 |
| **Norm. [5-^13^C]glutamate** | 2.61 ± 0.8 | 4.01 ± 1.4 | 0.78 ± 0.4 | 2.40 ± 0.7 |
| **Norm. [1-^13^C]acetylcarnitine** | 4.22 ± 1.1 | 5.16 ± 2.5 | 0.90 ± 0.5 | 2.99 ± 0.8 |
| **PCr/ATP ratio** | 2.23 ± 0.3 | 1.89 ± 0.3 | 2.45 ± 0.3 | 2.11 ± 0.1 |
| **ΔG_~ATP_ (kJ/mol)** | -64 ± 3 | -59 ± 3 | -65 ± 2 | -65 ± 2 |

Supplemental table 1: Experimental parameters presented as mean ± SD
